# Helminth-mediated disease tolerance in TB: A role for microbiota?

**DOI:** 10.1371/journal.ppat.1009690

**Published:** 2021-07-15

**Authors:** Danielle Karo-Atar, Nargis Khan, Maziar Divangahi, Irah L. King

**Affiliations:** 1 Meakins-Christie Laboratories, Department of Microbiology and Immunology, McGill University Health Centre, Quebec, Canada; 2 Meakins-Christie Laboratories, Departments of Medicine, Microbiology and Immunology, Pathology McGill University, McGill International TB Centre, McGill University Health Centre, Quebec, Canada; 3 McGill Interdisciplinary Initiative in Infection and Immunity, Montreal, Quebec, Canada; Geisel School of Medicine at Dartmouth, UNITED STATES

## Introduction

**Fig 1:**
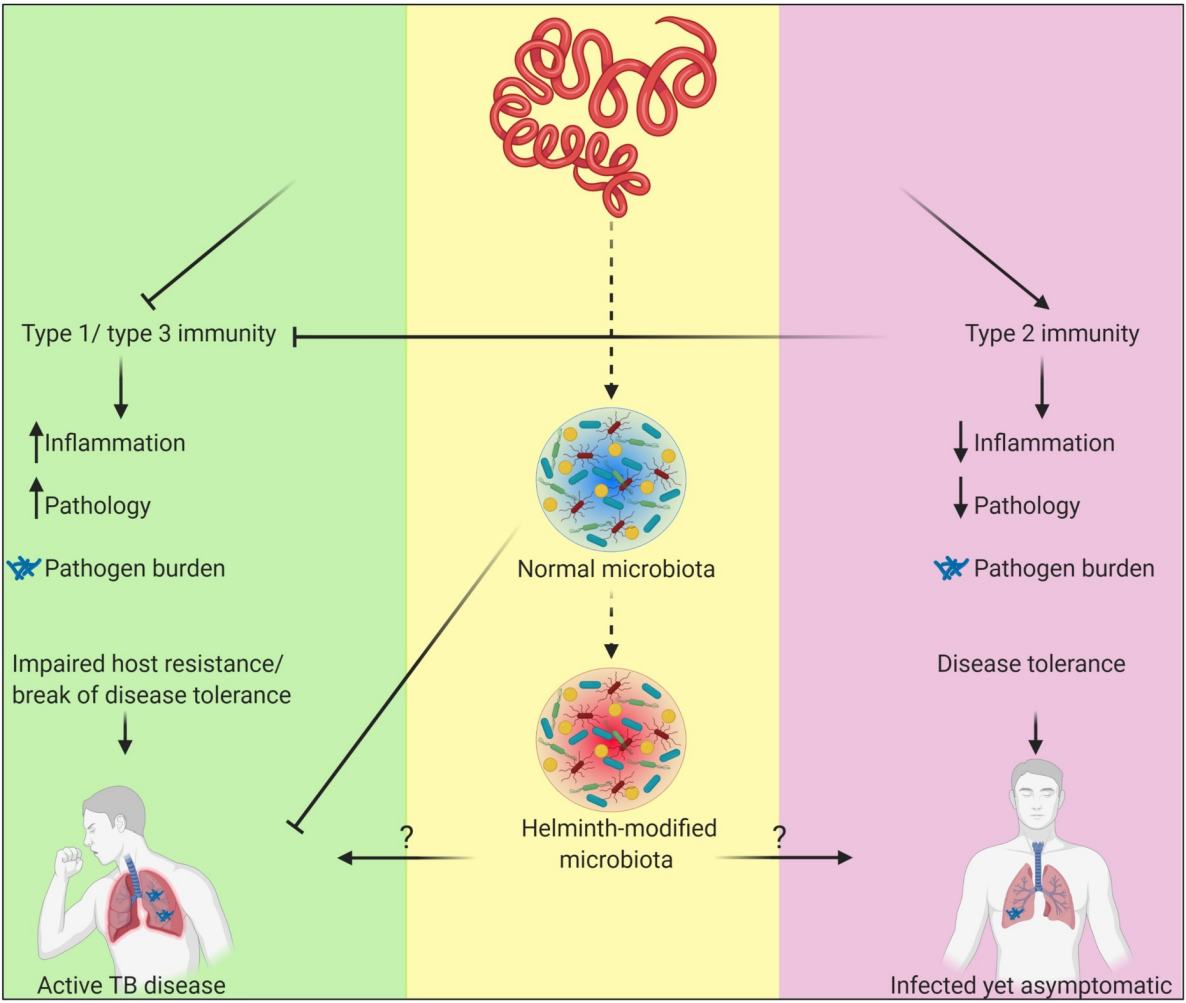
Helminth infections, eliciting robust type 2 immune responses, might contribute to *Mtb* disease tolerance by inhibiting type 1 and type 3 immune responses, thus reducing inflammation and pathology while maintaining bacterial burden. An alternative, but not mutually exclusive, possibility is that helminth-mediated changes to the gut microbiota shape TB outcomes. The robust regulatory capacity of the gut microbiota (via immune suppression, metabolite processing, and niche competition) is an appealing mechanism to explain the contradicting data regarding the exact role of helminth infections in TB disease progression and disease tolerance in asymptomatic infected patients. This figure was created with BioRender.com. *Mtb*, *Mycobacterium tuberculosis*; TB, tuberculosis.

Intestinal helminth infections are most prevalent in peri-equatorial regions of the world and have an overlapping geographical distribution with *Mycobacterium tuberculosis* (*Mtb*) infection—the causative agent of tuberculosis (TB). Importantly, approximately 40% of TB patients are asymptomatically infected with helminth parasites. While experimental and epidemiological evidence suggest that helminth infections alter the course of TB, other studies do not support this link [1]. Although the direct immunomodulatory effects of helminth infections on adaptive host immunity have been studied extensively, these can only partially explain the complex nature of helminth–TB interactions. Indeed, the potent immunomodulatory abilities of helminths may even reduce TB-associated tissue pathology [1] and contribute to disease tolerance [2]. However, helminth infections also induce changes to the gut microbiota that can have a systemic impact on heterologous infectious diseases [3]. Given previous studies demonstrating that the gut microbiota can shape disease tolerance to pulmonary infections [4], here, we discuss the current understanding of how the gut microbiota impacts TB and posit that helminth-mediated changes to this vast microbial community may contribute to the clinical course of TB in co-endemic regions (Fig 1).

## What is the impact of helminth infection on TB progression?

Infection with *Mtb* results in various clinical outcomes ranging from complete bacterial clearance or asymptomatic infection to active TB. The spectrum of this disease is largely dictated by 2 unique, but not mutually exclusive, host defense strategies: host resistance and disease tolerance. Host resistance to *Mtb* results in a decrease or elimination of the pathogen, an outcome that may result in irreversible, tissue damage. In contrast, disease tolerance pathways are engaged in controlling tissue damage rather than altering pathogen load [2]. While this latter strategy promotes host health and survival, it also leads to chronic infection. As 90% to 95% of exposed individuals remain asymptomatic, disease tolerance may be the most prevalent form of host defense against *Mtb* infection. However, a fraction of TB patients (5% to 10%) still maintain a lifetime risk of developing active disease. Thus, *Mtb* has coevolved with humans to achieve an evolutionary trade-off that infrequently compromises host health for survival. While it is unclear if the development of active TB results from a breakdown of host resistance and/or disease tolerance, we have recently shown in a preclinical animal model of TB that T cells play a key role in disease tolerance in TB [5]. Importantly, several additional factors have been linked to the progression from asymptomatic infection to active TB, including coinfection with helminths [6].

Several epidemiologic studies have established an association between TB progression and helminth infection [1,7,8]. However, the mechanistic rationale for this association is largely based on the fact that helminths induce a type 2 immune response, conventionally thought to be detrimental in TB. Helminth-induced type 2 immunity involves robust production of interleukin (IL)-4, IL-5, and IL-13 by CD4+ T helper type 2 (Th2) cells and type 2 innate lymphoid cells (ILC2s). Type 2 cytokines inhibit the generation of interferon gamma (IFNγ) and IL-17–producing T cells that are classically associated with resistance to TB. Indeed, coincident hookworm infection has been shown to suppress *Mtb*-specific T helper type 1 (Th1) and T helper type 17 (Th17) responses with an increase in regulatory T cells (Tregs) and Th2 cells in infected, asymptomatic patients [9]. However, O’Shea and colleagues found no impact of coincident hookworm infection on progression from latent to active TB [10], and McLaughlin and colleagues recently showed that *Mtb*-specific Th1 cytokine production capacity is maintained in helminth-infected individuals [11]. In addition, IL-4 and IL-13 signals promote alternative activation of macrophages, the primary cell type infected by *Mtb*, which may prevent sterile immunity, but also limit dissemination to peripheral organs [12]. By contrast, other studies have shown that helminth infection can be protective during the early stages of *Mycobacterium bovis* BCG infection [13]. Therefore, definitive data that these parasites promote progression from asymptomatic TB to active disease via T-cell immunomodulation are lacking. Alternatively, the immunoregulatory power of helminths may promote disease tolerance to TB. Support for this hypothesis is based on the ability of helminth infections to influence other diverse lung diseases. For instance, mice chronically infected with helminths are less prone to allergic airway inflammation and show reduced lung pathology by eliciting more Tregs [14]. Consistently, Tregs have been shown to induce better protection in chronic *Mtb*-infected mice by reducing lung pathology without any impact on bacterial burden [15]. Taken together, the outcomes of helminth–TB coinfected individuals may not be simply explained by an imbalance of Th1/Th2 cells. Many other factors might contribute to this complex heterologous infection including the timing of coinfection, anatomical location of the helminth, parasite load, or additional immune-regulatory factors such as the intestinal microbiota. Although these studies have led to important advancements in our understanding of *Mtb*–helminth coinfection, a more holistic approach involving the investigation of the intestinal microbiota in these conditions may shed new light on this complex interaction and resolve discrepant findings.

## What is the impact of helminths on commensal microbes and concurrent infections?

Many helminth species cohabitate with a vast collection of microbes (bacteria, viruses, and protozoa, aka, the microbiota) within the intestinal lumen. As such, the intestinal microbiota and helminths share the agenda of avoiding their expulsion from the mammalian gut. Thus, both have evolved mechanisms to modulate host immunity. Further, helminths are able to shape the intestinal microbiota via antimicrobial activity of their excretory–secretory products or modulation of host-derived antimicrobial peptides [16].

While in animal models, helminth infections have been shown to increase microbial diversity, data from human studies are more complex. Several studies assessing helminth-induced intestinal microbial changes have indicated an increase in microbial diversity and abundance, while others report no significant changes [17]. Nevertheless, the most common feature of worm infections is increased abundance of *Lactobacilli* species, which are capable of inducing host regulatory responses [16]. More specifically, intestinal helminths were shown to promote *Salmonella* coinfection by altering the intestinal metabolome. In addition, by using a fecal transplant approach, Zaiss and colleagues demonstrated that feces from *Hpb-*infected mice is enriched in short-chain fatty acids (SCFAs) and can reduce the severity of allergic lung inflammation, likely via the enhancement of Treg cell differentiation [3]. Indeed, SCFAs have also been shown to modulate host immunity to TB by directly reducing the secretion of inflammatory cytokines in peripheral blood monocytes [18]. In coinfection models, mice infected with *Hpb* had reduced respiratory syncytial virus (RSV) viral load and lung pathology in a microbiota-dependent manner [19]. Taken together, helminth-induced changes to the intestinal microbiota are an intriguing culprit that may modulate *Mtb* infection outcomes.

## Can microbiome alterations regulate TB progression?

Several studies indicate that changes to the microbiota modulate both host susceptibility to initial *Mtb* infection and the progression from asymptomatic to active disease [20]. Using a mouse model of antibiotic treatment to eliminate the intestinal microbiota, we and others found that changing the gut biodiversity compromised innate immunity to aerosol *Mtb* challenge [21–23]. Similarly, Majlessi and colleagues showed that intestinal *Helicobacter hepaticus* infection led to dysbiosis and an increase in *Mtb* burden [24]. Several clinical studies have also indirectly implicated the intestinal microbiota in promoting TB progression. In one study, *Mtb*-infected, asymptomatic patients with the presence of *Helicobacter pylori* in their gut flora were less likely to develop active TB disease, while another study showed that the commensal-associated metabolite, indole-3-propionic acid, exhibited antitubercular activity [21]. Taken together, these studies indicate that diverse perturbations to the intestinal microbiota regulate host susceptibility to *Mtb* infection. Whether helminth-associated intestinal microbiota alterations impact TB progression in coinfected individuals has not been addressed to date.

## Summary and conclusions

*Mtb* and helminth infections are co-endemic in major areas of the world, together affecting more than a quarter of the global population. In many cases, coinfected individuals exhibit altered TB disease progression, yet the exact role of helminth infections in TB outcomes highlight an important knowledge gap. In this Pearl, we suggest helminth-associated intestinal microbiota modulation as a potential mechanism underlying disease tolerance to *Mtb* infection or, at the very least, confound studies examining the impact of helminth infection on TB outcomes. Thus, investigating changes in the composition and/or functional output of the intestinal microbiota, with its far-reaching regulatory capacity (via immune suppression, metabolite processing, and niche competition), is needed to determine the relative contribution of diverse intestinal residents on *Mtb* infection. To this end, several approaches can be taken including the transfer of helminth-modified microbiomes in *Mtb* infection models, the use of *Mtb*/helminth coinfection models in germ-free mice, and critical microbiome analysis of TB patient cohorts before and after deworming treatment. These studies could advance our understanding of TB progression and pave the way toward designing more effective vaccines.
